# Deep Learning-Based Security Verification for a Random Number Generator Using White Chaos

**DOI:** 10.3390/e22101134

**Published:** 2020-10-06

**Authors:** Cai Li, Jianguo Zhang, Luxiao Sang, Lishuang Gong, Longsheng Wang, Anbang Wang, Yuncai Wang

**Affiliations:** 1 Key Laboratory of Advanced Transducers and Intelligent Control System, Ministry of Education, Taiyuan University of Technology, Taiyuan 030024, China; licai0759@link.tyut.edu.cn (C.L.); sangluxiao0134@link.tyut.edu.cn (L.S.); gonglishuang0122@link.tyut.edu.cn (L.G.); wanglongsheng@tyut.edu.cn (L.W.); wanganbang@tyut.edu.cn (A.W.); 2 College of Physics and Optoelectronics, Taiyuan University of Technology, Taiyuan 030024, China; 3 Guangdong Provincial Key Laboratory of Photonics Information Technology, Guangzhou 510006, China; 4 School of Information Engineering, Guangdong University of Technology, Guangzhou 510006, China

**Keywords:** deep learning, security analysis, random number generator, white chaos, semiconductor laser, predictive model

## Abstract

In this paper, a deep learning (DL)-based predictive analysis is proposed to analyze the security of a non-deterministic random number generator (NRNG) using white chaos. In particular, the temporal pattern attention (TPA)-based DL model is employed to learn and analyze the data from both stages of the NRNG: the output data of a chaotic external-cavity semiconductor laser (ECL) and the final output data of the NRNG. For the ECL stage, the results show that the model successfully detects inherent correlations caused by the time-delay signature. After optical heterodyning of two chaotic ECLs and minimal post-processing are introduced, the model detects no patterns among corresponding data. It demonstrates that the NRNG has the strong resistance against the predictive model. Prior to these works, the powerful predictive capability of the model is investigated and demonstrated by applying it to a random number generator (RNG) using linear congruential algorithm. Our research shows that the DL-based predictive model is expected to provide an efficient supplement for evaluating the security and quality of RNGs.

## 1. Introduction

Random number generators (RNGs) are extensively applied in the field of cryptography and security communications that require fast and trusted random numbers [1]. So far, there are two types of RNGs—deterministic random number generators (DRNGs) and non-deterministic random number generators (NRNGs). The output sequence of a DRNG is generated with a deterministic algorithm and a provided seed. Despite its good statistical characteristic, the DRNG is not suitable for information security applications, because the deterministic pattern of the DRNG may be identified by adversaries, which incurs malicious attacks and causes the destruction of security system, as in [2,3,4,5]. On the contrary, a NRNG produces the random sequence by using physical entropy sources, such as electrical noise [6,7,8], quantum fluctuations [9,10,11,12] and chaotic semiconductor lasers [13,14,15]. In particular, an ultra-fast NRNG using white chaos was proposed and demonstrated in [16,17], which has significant potential for improving the information security and securing the communications. However, any NRNG should not be assumed to be fully trusted or secure by default in the real world, because the presence of environmental noise or the unideal characteristics of the physical devices that construct the entropy sources may compromise the integrity of NRNGs [15,18]. Therefore, we deem security evaluation for random numbers necessary. 

The security analysis of RNGs is an important issue, since the security of cryptographic systems depends on the randomness and unpredictability qualities of RNGs. In most studies, the randomness of random numbers is evaluated by using standard statistical test suites, such as NIST Special Publication 800-22 [19], AIS 31 [20], Diehard [21], and TestU01 [22], which can detect whether there are obvious statistical defects among random numbers. However, limited by the ability of pattern recognition and data analysis, the standard statistical tests have shown the insufficiency in the security evaluation of RNGs. For instance, some DRNGs with good randomness can pass most statistical tests successfully, although there are weak but inherent correlations among them [23]. It is necessary to further investigate the security analysis methods and tools for RNGs.

Recently, deep learning (DL) has attracted great attention because of its powerful capability in recognizing patterns and discovering intricate structures in large data sets [24]. Considering its strength in learning nonlinear manifolds of data [25], researchers have explored several security analysis methods of random numbers by DL. In [26,27], the authors implemented feedforward neural network (FNN) structures for detecting hidden patterns among pseudo-random numbers from DRNGs. Wen et al. [28] constructed a long short-term memory (LSTM)-based DL to evaluate the randomness of a new DRNG that consists of both regular DRNGs and a physical unclonable function (PUF). However, the above works did not study and prove the performance of DL models in detecting inherent correlations among data. Yang et al. [29] proposed a novel min-entropy estimation method based on DL models composed of a FNN and a recurrent neural network (RNN) to estimate min-entropy of entropy sources of RNGs. Unfortunately, the estimator is easily given to overestimation for data with subtle correlations. In addition, Zhu et al. [30] improved the min-entropy estimation on time-varying data by applying a change detection method to a FNN-based estimator. Truong et al. [18] developed a recurrent convolutional neural network (RCNN)-based predictive model, which detected prominent inherent correlations of deterministic noise sources in a quantum random number generator. Although DL has promising applications in evaluating the quality of random sequences, there are few studies on the security analysis for NRNGs based on white chaos by deep learning.

In this paper, a DL-based predictive analysis is proposed to analyze the security of RNGs. In particular, the temporal pattern attention (TPA)-based DL model is employed to detect hidden correlations that may exist among the long random sequence from RNGs, and then predict the next random number, based on observed random numbers in an input sequence. Next, we investigate the learning capability of the DL model in detecting deterministic correlations, which is applied to the liner congruential DRNG with different periods. In addition, compared with the existing related works, the performance of the model is further evaluated on the prediction accuracy and the length of the training data. Finally, we implement a white chaos-based NRNG, and analyze the security of the NRNG by DL. In particular, the predictive model is used to analyze the security of the data extracted from both stages of the NRNG: the output of a chaotic external-cavity semiconductor laser (ECL) and the final output of the NRNG. Additionally, we investigate the reasons behind the advantage provided by DL.

## 2. Experimental Scheme

**Figure 1:**
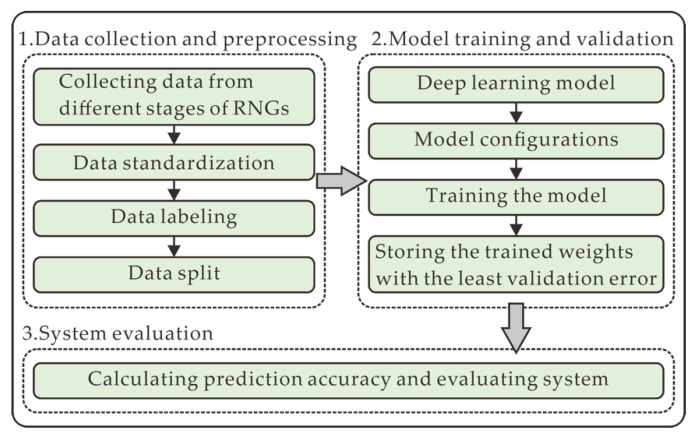
Experimental scheme for evaluating the security of RNGs, which comprises data collection and preprocessing, model training and validation, and system evaluation.

In this section, the overall experimental scheme is illustrated in Figure 1, mainly comprising data collection and preprocessing (Section 2.3), model training and validation (Section 2.5), and system evaluation (Section 2.6). In the data collection and preprocessing, the datasets are firstly collected from different stages of RNGs that are described in Section 2.1 and Section 2.2. Then, the raw data is standardized in the form of *N*-bit integers, labeled in the supervised learning approach, and split into three sets including training set, validation set, and test set. In the model training and validation, the predictive DL model is provided and depicted in Section 2.4. After its parameters are configured, the model is trained and validated in this process. In system evaluation, the performance of the model, and the security of the data are evaluated by the prediction accuracy.

### 2.1. White Chaos-Based NRNG Setup

**Figure 2:**
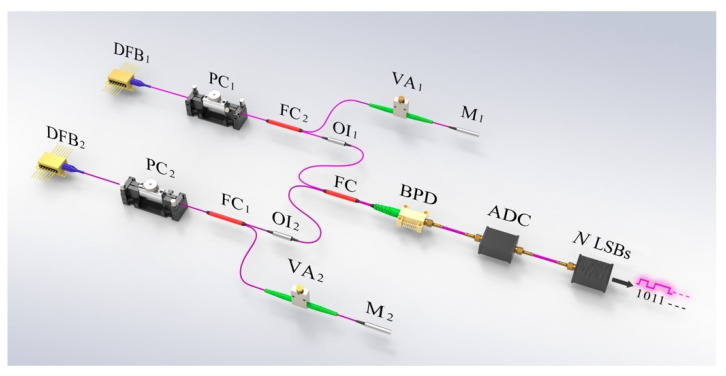
The structure of a NRNG based on white chaos. DFB_1,2_: distributed feedback semiconductor laser; PC_1,2_: polarization controller; FC_1,2_, FC: fiber coupler; OI_1,2_: optical isolator; VA_1,2_: variable attenuator; M_1,2_: fiber mirror; BPD: balanced photo-detector; ADC: analog-to-digital converter; LSBs: least significant bits.

The structure of a white chaos-based NRNG consists of an entropy source and entropy extractor, as illustrated in Figure 2 and described in detail in [16]. The generation of white chaos [17], a physical process, can be taken as an entropy source for the NRNG. Two ECLs are introduced into the entropy source, each of which contains a distributed feedback semiconductor laser (DFB) with optical feedback. It is noted that the optical feedback is implemented by a feedback external cavity composed of the laser facet and a fiber mirror. In the feedback cavity, a polarization controller is inserted behind each laser to adjust the polarization of the feedback light, and a variable attenuator is placed in front of the corresponding mirror to tune up the intensity of the feedback light. After optical isolators, the outputs of both lasers are coupled by a 3-dB fiber coupler, and then two optical signals are injected into a balanced photo-detector, in which both identical photodetectors and an electronic circuit are integrated to detect the heterodyne signal. For the entropy extractor, the heterodyne signal is quantized by an 8-bit analog-to-digital converter (ADC), and then the random numbers are generated by selecting *N* consecutive least significant bits (LSBs) at each sampled value.

Experimentally, the lasers DFB_1_ and DFB_2_, respectively operating at bias currents of 15.6 mA and 15.3 mA, have threshold currents of 10.9 mA and 11.1 mA, respectively. The center wavelengths of DFB_1_ and DFB_2_ are 1549.73 nm and 1549.62 nm, respectively. The feedback strength is set to −8.1 dB for ECL_1_ and −7.9 dB for ECL_2_. In addition, the feedback delays of both ECLs are 91.7 ns and 91.9 ns, respectively. With these parameters of the entropy source, the white chaos is generated by optical heterodyning. After quantization with the 8-bit ADC, a 320 Gb/s white chaos-based NRNG is realized by selecting 4 LSBs at 80-GHz sampling rate.

For security analysis of the NRNG, we investigate the quality of data collected from the output of the ECL_1_ and the final output of the NRNG. These are done by applying a novel DL model to data extracted at both stages of the NRNG. Note that the security of ECL_1_ is only evaluated due to the similarity of ECLs.

### 2.2. DRNG Setup

To demonstrate the robustness of provided DL model, a linear congruential random number generator (LC-RNG) [31], a typical deterministic mechanism used in many software platforms, is introduced in our experiments. Because a benefit of the LC-RNG is that with appropriate choice of parameters, the period is known and long. The algorithm of LC-RNG is described by recurrence relation:

(1) $$X_{n+1}=\left(aX_{n}+c\right)\mathrm{mod}M,$$

where *X* represents the sequence of random numbers, and *M*, *a*, and *c* are integer constants, which represent the modulus, multiplier, and increment of the generator, respectively. With correctly chosen parameters, the period of the random values is equal to *M* for any seed. The generation of pseudo-random numbers will occur if: (1) *M* and *c* are relatively prime, (2) *a* − 1 is divisible by all prime factors of *M*, and (3) *a* − 1 is divisible by 4 if *M* is divisible by 4. In our experiments, we collected the pseudo-random sequences generated by LC-RNG with *a* = 25214903917, *c* = 1 and *M* ∈ (2^24^, 2^26^, 2^28^, 2^30^). It is necessary to study the predictive capability of the DL model in discovering inherent and intricate dependencies.

### 2.3. Data Collection and Preprocessing

In the data acquisition stage, we collect several datasets extracted at different stages of the introduced NRNG, and LC-RNG. At each stage or period of RNGs, 200 million raw random numbers are gathered and standardized in the form of *N*-bit integers. Out of these, 40%, 10%, and 50% are used for training, validating, and testing the provided DL model, respectively. To assess the consistency of the learning performance of the model, the test dataset is divided into five sub-test datasets, and each of them comprises twenty million random numbers.

**Figure 3:**
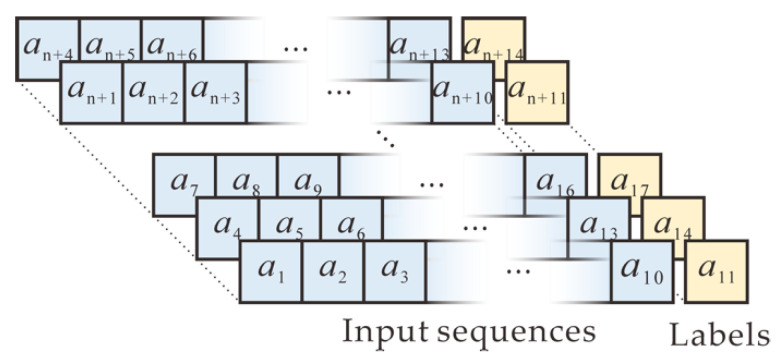
Data preprocessing in a conventional approach where 10 consecutive adjacent numbers within the random sequence are used as one input sequence, whereas the next number after the input sequence is used as the output (label). The new sequence and corresponding output are updated by shifting three positions in the dataset.

Our task is to learn hidden correlations among the random numbers of RNGs and predict the next number, based on observed random numbers in an input sequence. Therefore, supervised learning with a neural network is performed in data preprocessing. The sequence of collected random numbers is arranged in a conventional approach, as shown in Figure 3. Specifically, ten consecutive adjacent numbers within the random sequence are used as one input sequence, whereas the next number after the input sequence is used as the output (label). Next, the sequence is shifted by three positions and is updated as another input. Similarly, the next number after the new input sequence also is used as the new output. The shifting process continues until all input sequences and corresponding outputs are generated. In addition, the neural network is trained and tested in the processed datasets in the format of pairs.

### 2.4. Deep Learning Model

Since the output of RNGs is a typical time series, we prefer to focus on recurrent neural networks (RNNs) [32], which are typical deep neural networks designed for sequence modeling. Nevertheless, simple RNNs are subject to the problem of vanishing gradients during training, and have difficulty discovering deterministic correlations [33]. In recent years, long short-term memory (LSTM) and gated recurrent unit (GRU), two popular variants of RNNs, have overcome the limited shortcoming of discovering long-term dependencies to some extent, and have achieved success in various applications [34,35,36]. To further solve time series prediction problems, some researchers have introduced attention mechanisms into deep neural networks [37,38,39]. Inspired by [40], a temporal pattern attention (TPA)-based LSTM is applied to the DL model to capture inherent correlations among random numbers in this paper. Compared with the typical attention mechanism, the provided TPA mechanism can learn the hidden correlations in the intricate time series data with advantage.

**Figure 4:**
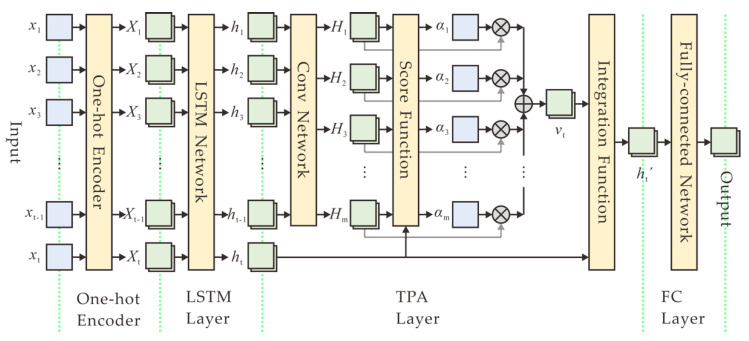
Deep learning model based on temporal pattern attention, which consists of a one-hot encoder, a LSTM layer, a temporal pattern attention (TPA) layer, and a fully connected (FC) layer.

The structure of a TPA-based DL model mainly consists of a one-hot encoder, a LSTM layer, a TPA layer, and a fully connected (FC) layer, as depicted in Figure 4. Specifically, after data collection and preprocessing, ten *N*-bit numbers are firstly encoded into one-hot vectors, each of which is a binary vector that has all zeros values except a significant value used to distinguish different numbers. Then the encoded vectors are sequentially fed to a LSTM layer with 256 output size, which can output the hidden states corresponding to each time step in an input sequence. Afterwards, the output of the LSTM layer is connected to the TPA layer. The attention layer analyzes the information across all previous time steps and selects relevant information to help generate the output. Finally, the attention output configured to size 256 goes to a fully connected (FC) layer, with a softmax activation function because of the multi-classification problem. The output size of the FC layer is configured to 2*^N^*, which is the number of all possible *N*-bit numbers in predicting the next value.

In the temporal pattern attention mechanism [40], given the previous LSTM hidden states *H* = (*h*_1_, *h*_2_, …, *h*_t-1_) ∈ ℝ*^m^*^×(*t-*1)^, a convolutional neural network (CNN) is used to improve the predictive performance of the model by employing CNN filters on the row vectors of *H*. The CNN has *k* filters, each of which has length of *T*. In addition, the CNN with a rectified linear unit activation function yield *H*^C^ ∈ ℝ*^m^*^×*k*^, where *H_i_* denotes the convolutional value of the *i*-th row vector of *H*. Then, the context vector is calculated as a weighted sum of row vectors of *H*^C^. The score function ƒ is defined to evaluate relevance between *H_i_* and *h_t_*:

(2) $$f\left(H_{i},h_{t}\right)={\left(H_{i}\right)}^{Τ}W_{a}h_{t},$$

where *h_t_* is the present state of the LSTM output, and *W_a_* ∈ ℝ*^k^*^×*m*^. The attention weight *α_i_* is realized by introducing a sigmoid activation function:

(3) $$\alpha_{i}=sigmoid\left(f\left(H_{i},h_{t}\right)\right).$$

To obtain the context vector *v_t_* ∈ ℝ*^k^*, the row vectors of *H*^C^ is weighted by *α_i_*:

(4) $$v_{t}=\sum_{i=1}^{m}\alpha_{i}H_{i}.$$

Finally, we integrate *v_t_* and *h_t_* to yield the output of the attention layer,

(5) $$h_{t}^{\prime }=W_{h}h_{t}+W_{v}v_{t},$$

where *W_h_* ∈ ℝ*^m^*^×*m*^, *W_v_* ∈ ℝ*^m^*^×*k*^.

In our experimental model, we set the time steps of an input sequence *t* = 10, the output size of the LSTM *m* = 256, the number of filters *k* = 256, and the length of a filter *T* = 10.

### 2.5. Model Training and Validation

In addition to the appropriate DL model as a solution for maximizing the probability of predicting the next value successfully, the DL model needs to be configured with several key parameters. The predictive model is regarded as a solution to a multi-classification problem, so the cross-entropy is used as an objective function, which can calculate the bias between the labels and the predicted values. In addition, an Adam optimizer [41] with a manually set 0.0005 learning rate is introduced to minimize the objective function during training phase. A batch of examples of size 256 is fed into the model during both of the training and the testing. The maximum number of epochs is set to 200, and the training is discontinued once the validation error stops decreasing after 5 successive epochs. The validation error and corresponding trained weights are recorded in the training phase, and the trained weights with the least validation error are used as the final trained weights for evaluating the corresponding test set.

### 2.6. System Evaluation

Not only the learning capability of the predictive model, but also the security of the data is evaluated by comparing the probability of correct prediction, *P_pred_*, against the baseline probability, *P_b_*, which is the highest probability of guessing a variable in the data. For a DL model, *P_pred_* is the probability of predicting the eleventh number correctly in the test set, according to the preceding ten consecutive numbers. That is, *P_pred_* is a percentage of all the correct predictions out of the total number of test predictions,

(6) $$P_{pred}=\frac{N_{T}}{N_{T}+N_{F}}\times 100\%,$$

where *N_T_* is the number of correct classifications, and *N_F_* is the number of incorrect classifications. The baseline probability *P_b_* is related to the minimum entropy of the distribution from which a random value is generated. In NIST Special Publication 800-90B [42], the min-entropy of an independent discrete variable *X* that takes values from a set *A* = (*x*_1_, *x*_2_, …, *x_k_*) with probability *Pr*(*X* = *x_i_*) = *p_i_*, for *i* = 1, …, *k* is described as:

(7) $$H=\log_{2}\underset{1\leq i\leq k}{\max}p_{i},$$

(8) $$=\log_{2}\left(P_{b}\right).$$

If a random variable has min-entropy *H*, the probability of observing any specific value for *X* is no greater than *P_b_*, which is why it is considered to be the baseline probability. For instance, an *N*-bit random number from datasets extracted at a certain stage of the NRNG or LC-RNG has a uniform probability distribution, which means that the highest probability of guessing the output of RNGs is 1/2*^N^*. If the DL-based predictive model could give a higher prediction probability compared to the baseline probability, there exist hidden correlations in the data from the corresponding stage of RNGs. Contrarily, little is learned by the model, and the random numbers have strong resistance against the predictive DL model. On the other hand, compared with the statistical property tests, the performance of the predictive model is studied by learning deviations in the data with different level of complexity. 

## 3. Experimental Results

In this paper, the DL model is implemented based on Keras and the backend of TensorFlow with Python language. In addition, all experiments are performed on a Windows 10 system with an Intel i9 10900X CPU and two NVIDIA RTX 2080Ti GPUs.

**Figure 5:**
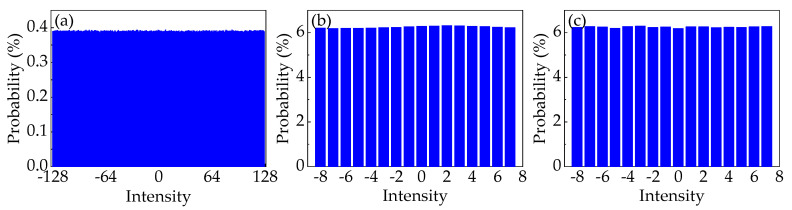
Distribution of standardized numbers from RNGs with different stages. (**a**–**c**) represent the probability distribution of the data from the output of LC-RNG with the period of 2^24^, the output of the ECL_1_, and the output of the NRNG, respectively.

For the first scenario, we investigate the learning capability of the predictive model, which is applied to random numbers collected from the LC-RNG with different periods. Before that, the probability distribution of the 8-bit random numbers at different periods is measured to calculate the corresponding baseline probability. Specifically, the raw values of the intensity of the temporal waveforms are standardized in the form of 8-bit integers between −128 and 127 to generate the corresponding histogram. From these histograms, the 8-bit standardized numbers at different stages are basically subject to the same uniform probability distribution. That is, the baseline probability is 0.39%. In particular, the probability distribution of random integers from LC-RNG with the period of 2^24^ is shown in Figure 5a. 

**Figure 6:**
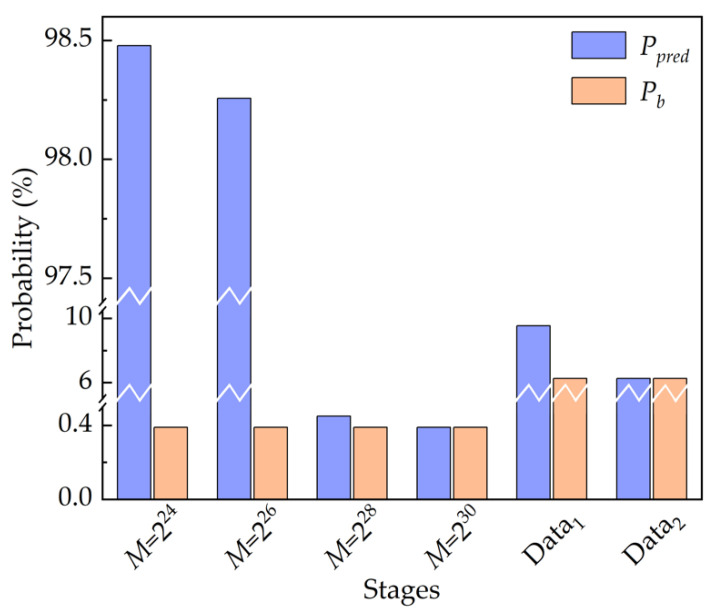
Prediction performance of the deep learning-based predictive model at different stages of LC-RNG and the white chaos-based NRNG.

Then, the model shows the predictive capability in learning the inherent and long-term correlations among pseudo-random numbers, as manifested in Figure 6. The model achieves 98.478 ± 0.07%, 98.256 ± 0.06%, 0.45 ± 0.01%, 0.39 ± 0.01% accuracy in predicting the next random number given precedent consecutive 10 numbers when the period of the LC-RNG, *M*, is 2^24^, 2^26^, 2^28^, 2^30^, respectively. Please note that the seed for generating pseudo-random numbers in a training set is different from that in the corresponding test set. Evidently, the probability of correct prediction by the model, *P_pred_*, surpasses the baseline probability, *P_b_*, when the length of the training set exceeds the period of LC-RNG, i.e., *M* is less than 2^28^. In addition, the provided model still has *P_pred_* better than *P_b_* by more than 6 standard deviations, even if *M* is 2^28^, which is much larger than the length of the training set. Meanwhile, *P_pred_* decreases when *M* increases given the same size of training set. When *M* is 2^30^ or larger, *P_pred_* is approximately equal to *P_b_*. It could be that the datasets with higher level of complexity make the model more difficult to detect the correlations among random numbers.

To substantiate the performance of the attention-based DL model, the NIST Special Publication 800-22 test suite [19] is employed for evaluating random numbers extracted from the LC-RNG. The test suite is a standard statistical package composed of 15 tests to evaluate the security of random values generated by any RNG. These tests reporting *P*-values within the range of 0.01–1.00 are considered to accept the hypothesis that the tested random numbers exhibit no characteristics of order or structure. The test results of random numbers with different periods are shown in Table 1. The number of passing tests increases as *M* increases. The test suite can detect deviations among the data when *M* is 2^24^, 2^26^, respectively. However, the random numbers can pass 15 tests of the test suite when *M* is 2^28^, 2^30^, respectively. Compared with the corresponding results from Figure 6, the DL model still achieves a higher prediction probability than the baseline probability, when random numbers with the period of 2^28^ can pass the NIST test suite successfully. Briefly, the DL-based predictive model has the advantage in detecting correlations among random numbers to some extent, compared to the results of the NIST test suite.

Furthermore, the learning performance of the TPA-based model is further evaluated on the prediction accuracy and the length of the sequence used for training, compared with the existing typical DL methods of evaluating the security of RNGs, including RNN-based model [29], FNN-based model [26,27,29], RCNN-based model [18]. These models are trained and tested using the same experimental strategy (see Section 2.3, Section 2.4, Section 2.5 and Section 2.6). The configuration of the models we compared is shown in Table 2 and described in detail in [18,26,27,29]. It is noted that the hyperparameters of the models are modified and optimized to fit the provided strategy, and improve the prediction accuracy. To evaluate the performance, the average prediction accuracy in the five test subsets is taken as the evaluation criterion.

On the prediction accuracy, these models are applied to twenty million datasets from LC-RNG with *M* ∈ (2^20^, 2^22^, 2^24^, 2^26^). The prediction results of these deep learning models on the LC-RNG with different periods are shown in Table 3. The baseline probability, *P_b_*, is still 0.39%, since 8-bit random numbers extracted from different periods follow the same uniform probability distribution. In Table 3, the simple RNN-based model has no advantage in detecting the intricate correlations among random numbers when *M* ≥ 2^22^. We speculate that the RNN-based model is subject to the problem of gradient disappearance during the training process, and has difficulty in discovering deterministic correlations. The FNN-based model and RCNN-based model can detect correlations in the data when *M* ≤ 2^26^, and give higher prediction accuracy than *P_b_*. However, the TPA-based model consistently achieves a prediction accuracy of more than 95% when *M* ≤ 2^24^, which is significantly better than the performance of other models. The model still detects the correlations, even though the length of the training set is less than *M*.

To compare the effectiveness of these models, we also investigate how the prediction accuracy depends on the length of the training data, *L*. We draw the prediction results of these models on the LC-RNG with the period of 2^24^, which are shown in Table 4. The RNN-based model with simple configuration still shows the weak learning capability when *L* increases. The performance of FNN-based model and RCNN-based model becomes better as *L* increases. These results show that the longer the length of the training set is, the higher the prediction accuracy. In addition, the FNN-based model performs better than others when *L* = 3.2 × 10^6^, because it consumes most computational resources (trainable parameters) among these models. The TPA-based model gives an obvious advantage in learning the correlations when *L* increases, compared with the performance of others. Specifically, given the same length of the training data, the model achieves higher prediction accuracy than other models when *L* ≥ 6.4 × 10^6^. As shown above, the performance of the predictive model is investigated and demonstrated in this scenario.

For the second scenario, we investigate the security of datasets extracted at different stages of the NRNG based on white chaos from the perspective of DL. Because of the retention of four LSBs in quantization of white chaos, the security of 4-bit data is evaluated in this scenario. Prior to this, the probability distribution of 4-bit datasets extracted at different stages of the NRNG is verified, as shown in Figure 5b,c. Obviously, the 4-bit integers at different stages are basically subject to the same uniform probability distribution. That is, the baseline probability is 6.25%. Then, the same procedure is used to learn the hidden correlations in the datasets, which are gathered from the output of the ECL_1_ (denoted as Data_1_) and the final output of the NRNG (denoted as Data_2_). The results of the prediction are also shown in Figure 6. For the ECL_1_ stage, the predictive DL model achieves 9.54 ± 0.05% accuracy, which obviously surpasses *P_b_* in guessing the next random value. For the final output of the NRNG, *P_pred_* is extremely close to *P_b_*, i.e., the provided model learns no patterns in the training dataset. For both stages of the NRNG, the results given the DL model are consistent with these of the NIST test suite in Table 1. In other words, the predictive model does as well as the NIST test suite in this scenario.

**Figure 7:**
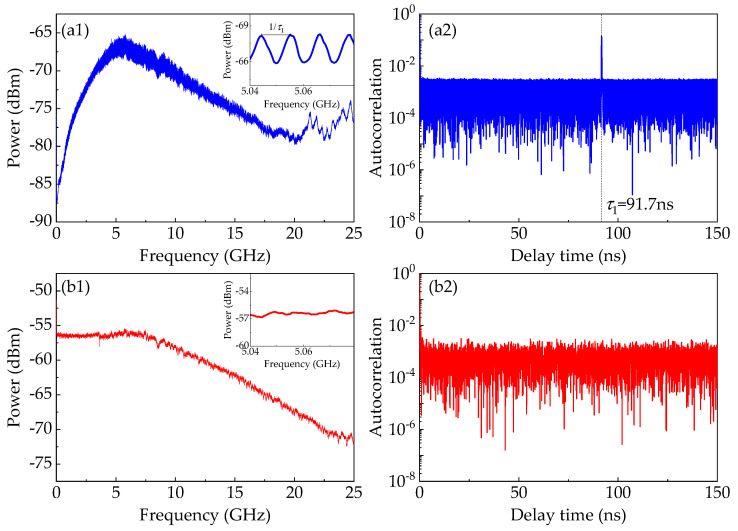
Temporal properties of the chaos of the ECL_1_ as well as the white chaos-based NRNG. (**a1**,**b1**) respectively represent the RF spectra of the chaos of the ECL_1_ and the white chaos. (**a2**,**b2**) respectively represent the autocorrelation traces of the chaos of the ECL_1_ and the white chaos.

To further investigate the reasons behind the advantage provided by the DL model, temporal properties of the white chaos of the NRNG as well as the chaos of the ECL_1_ are depicted in Figure 7, including the radio-frequency (RF) spectrum, and the autocorrelation function. The RF spectra of the chaos of the ECL_1_ and the white chaos are depicted in Figure 7a1,b1, respectively. For the spectrum map of the chaotic ECL_1_, a dominant peak approximately at the relaxation frequency can be clearly observed, which is detrimental to the bandwidth and flatness of chaos of ECL_1_ [17]. Furthermore, we can observe an obvious pattern of periodic modulation from the insert of Figure 7a1. Please note that the period equals the reciprocal of the feedback delay time. The periodic modulation is actually the time-delay signature (TDS) that destroys the unpredictability and randomness of entropy source. However, in Figure 7b1, the spectrum of the white chaos is flat and broadband, which is not subject to the dominant peak and the periodic modulation pattern. That is, the white chaos generated by optical heterodyning has the great potential in extracting high-speed and trusted random numbers. 

To examine the existence of TDS, we plot the autocorrelation traces of the chaos of the ECL_1_ and the white chaos, as depicted in Figure 7a2,b2, respectively. The autocorrelation trace of the chaotic ECL_1_ shows an apparent correlation peak at the feedback delay in Figure 7a2. We speculate that the retention of four LSBs still preserves the TDS in raw data, which precludes its use as a random number generator. By comparison, after optical heterodyning, the correlation trace of the heterodyne signal has no correlation peak in Figure 7b2, which indicates the elimination of such time-delay signature by heterodyning of two chaotic ECLs. In addition, other methods [43,44,45] of eliminating the TDS also significantly improve the randomness of RNGs.

In the predictive model, the CNN is introduced into the TPA mechanism. As demonstrated in [46], the CNN filters play a role of bases in the discrete Fourier transform (DFT), which is used to reveal significant temporal characteristics in the intricate time series. Originally, the frequency domain in DFT serves as a powerful representation for CNN to use in training and modeling [40]. The frequency-domain representation from CNN filters can reveal the signature of the time delay of the data from the chaotic ECL_1_ stage. Thus, we believe that TDS of the chaotic ECL_1_ causes the correlations among the data, and then gives the predictive model more chances to learn any temporal information among the data. For the white chaos, TDS is eliminated by heterodyning of two chaotic ECLs, and no characteristics are shown in the frequency domain. Evidently, the model cannot learn any temporal pattern in the training dataset collected from the final output of the NRNG, i.e., *P_pred_* ≈ *P_b_*. Therefore, the NRNG has the strong resistance against our predictive DL.

## 4. Discussion

It is surprising that the DL-based predictive model can perform our task quite meaningfully, as evidenced above. Specifically, the model can learn inherent correlations among random numbers, and gives obvious and consistent prediction accuracy better than the baseline probability in five sub-test sets, when *M* ≤ 2^28^. Note that random numbers with the period of LC-RNG of 2^28^ can pass the NIST test suite successfully. Additionally, the model can also detect deterministic patterns caused by TDS in the ECL_1_ stage of the NRNG. However, little is learned by the model when the period of LC-RNG is larger than 2^28^. The prediction ability of the model is limited by the basic DL architecture and its parameters, such as the length of the training set, the size of the input sequence, and so on. Apparently, the optimization of the parameters and the sophisticated and advanced neural networks can improve the prediction accuracy. 

On the other hand, it is essential to pursue higher prediction accuracy by using all technical methods. The higher probability of correct prediction indicates the more powerful capability of the model in detecting inherent correlations of random numbers. In cryptography and security communications, in order to avoid attacks by adversaries, RNGs ought to comply with more stringent test requirements, including the DL-based predictive models. 

## 5. Conclusions

In conclusion, a predictive analysis using DL based on TPA is proposed to evaluate the security of RNGs. The predictive model has powerful learning capability in detecting inherent correlations among random numbers, which is investigated and demonstrated by applying it to the LC-RNG with different periods. Compared with the existing related works, the learning performance of the model is further verified on the prediction accuracy and the length of the training data. After that, we analyze the security of data extracted at both stages of the NRNG based on physical white chaos. In particular, for the ECL_1_ stage, the model learns deterministic correlations among the dataset, and achieves higher accuracy than the baseline probability in guessing the next random number. After optical heterodyning of both chaotic ECLs and minimal post-processing are introduced, the predictive model detects no patterns in the data; this is the first work showing that the NRNG has the strong resistance against DL. By analyzing the temporal properties of both stages, we find that TDS, causing the inherent correlations among the data, is the key to be learned and detected by DL. Finally, we conclude that DL-based predictive model is expected to provide an efficient supplement for evaluating the security and quality of RNGs.

Even though we confirmed the powerful learning capability of our predictive model, it is still worthwhile further optimizing the predictive performance of the model, and deeply investigate the potential of DL in cryptanalysis of RNGs in the future. In addition, we will apply the advanced DL technologies to construct the predictors for entropy estimation of RNGs. Moreover, the DL-based predictors will be employed for real-time health testing of entropy sources of RNGs in our future work.

## Figures and Tables

**Table 1:** Results of NIST statistical test suite on the datasets at different stages of LC-RNG and the white chaos-based NRNG.

Statistical Tests	LC-RNG				NRNG	
	*M* = 2^24^	*M* = 2^26^	*M* = 2^28^	*M* = 2^30^	Data_1_	Data_2_
Frequency	Success	Success	Success	Success	Failure	Success
Block Frequency	Success	Success	Success	Success	Success	Success
Cumulative Sums	Success	Success	Success	Success	Failure	Success
Runs	Success	Success	Success	Success	Failure	Success
Longest Run	Success	Success	Success	Success	Success	Success
Rank	Success	Success	Success	Success	Success	Success
FFT	Failure	Success	Success	Success	Success	Success
Non-overlapping Template	Failure	Failure	Success	Success	Failure	Success
Overlapping Template	Success	Success	Success	Success	Success	Success
Universal	Success	Success	Success	Success	Success	Success
Approximate Entropy	Failure	Success	Success	Success	Failure	Success
Random Excursions	Success	Success	Success	Success	Success	Success
Random Excursions Variant	Success	Success	Success	Success	Success	Success
Serial	Failure	Success	Success	Success	Failure	Success
Linear Complexity	Success	Success	Success	Success	Success	Success
Total successful tests	11/15	14/15	15/15	15/15	9/15	15/15

**Table 2:** Model configuration of neural networks.

RNN-Based Model	FNN-Based Model	RCNN-Based Model
Input layer ^1^	Input layer ^1^	Input layer ^1^
RNN-256 + Tanh	FC-256 + Relu	CNN ^2^-64 + Relu + MP-2
FC-256 + Softmax	FC-256 + Relu	CNN ^3^-128 + Relu+ MP-2
/	FC-256 + Softmax	LSTM-128 + Tanh
/	/	FC-256 + Softmax

^1^ The input layer with a one-hot encoder; ^2^ the CNN with a filter length of 9; ^3^ the CNN with a filter length of 3.

**Table 3:** Prediction performance of the models on the LC-RNG with different periods.

Model	LC-RNG (Accuracy: %)			
	*M* = 2^20^	*M* = 2^22^	*M* = 2^24^	*M* = 2^26^
RNN-based model	44.42 ± 0.02	0.39 ± 0.01	0.39 ± 0.01	0.39 ± 0.01
FNN-based model	99.81 ± 0.01	88.62 ± 0.05	77.82 ± 0.05	1.88 ± 0.02
RCNN-based model	86.91 ± 0.05	61.45 ± 0.07	18.79 ± 0.06	1.93 ± 0.01
TPA-based model	99.86 ± 0.02	99.38 ± 0.06	95.53 ± 0.05	0.39 ± 0.01

**Table 4:** Prediction performance of the models on the LC-RNG with different length of training data.

Model	Length of Training Data (×10^6^)			
	1.6	3.2	6.4	8.0
RNN-based model	0.39 ± 0.01	0.39 ± 0.01	0.39 ± 0.01	0.39 ± 0.01
FNN-based model	0.39 ± 0.01	67.62 ± 0.05	74.65 ± 0.03	77.82 ± 0.05
RCNN-based model	0.39 ± 0.01	0.39 ± 0.01	10.37 ± 0.03	18.79 ± 0.06
TPA-based model	0.39 ± 0.01	1.03 ± 0.02	92.80 ± 0.03	95.53 ± 0.05

## Abbreviations

The following abbreviations are used in this manuscript:

DL	Deep Learning
NRNG	Non-deterministic Random Number Generator
DRNG	Deterministic Random Number Generator
TPA	Temporal Pattern Attention
ECL	External-Cavity Semiconductor Laser
RNG	Random Number Generator
FNN	Feedforward Neural Network
RNN	Recurrent Neural Network
RCNN	Recurrent Convolutional Neural Network
DFB	Distributed Feedback Semiconductor Laser
PC	Polarization Controller
FC	Fiber Coupler
OI	Optical Isolator
VA	Variable Attenuator
BPD	Balanced Photo-Detector
ADC	Analog-to-Digital Converter
LSBs	Least Significant Bits
LC-RNG	Linear Congruential Random Number Generator
LSTM	Long Short-Term Memory
GRU	Gated Recurrent Unit
FC	Fully Connected
CNN	Convolutional Neural Network
MP	Max-Pooling
RF	Radio-Frequency
TDS	Time-Delay Signature
DFT	Discrete Fourier Transform
